# Advancements in Home-Based Devices for Detecting Obstructive Sleep Apnea: A Comprehensive Study

**DOI:** 10.3390/s23239512

**Published:** 2023-11-30

**Authors:** Miguel A. Espinosa, Pedro Ponce, Arturo Molina, Vicente Borja, Martha G. Torres, Mario Rojas

**Affiliations:** 1 Institute of Advanced Materials for Sustainable Manufacturing, Tecnologico de Monterrey, Mexico City 14380, Mexico; a01339422@tec.mx (M.A.E.); mario.rojas@tec.mx (M.R.); 2 Faculty of Engineering, Universidad Nacional Autonoma de Mexico, Mexico City 04510, Mexico; vicente.borja@ingenieria.unam.edu; 3 Sleep Medicine Unit, Instituto Nacional de Enfermedades Respiratorias Ismael Cosio Villegas, Mexico City 14080, Mexico; torresfraga@iner.gob.mx

**Keywords:** sleep apnea detection, oximetry, actigraphy, respiratory effort, respiratory flow

## Abstract

Obstructive Sleep Apnea (OSA) is a respiratory disorder characterized by frequent breathing pauses during sleep. The apnea–hypopnea index is a measure used to assess the severity of sleep apnea and the hourly rate of respiratory events. Despite numerous commercial devices available for apnea diagnosis and early detection, accessibility remains challenging for the general population, leading to lengthy wait times in sleep clinics. Consequently, research on monitoring and predicting OSA has surged. This comprehensive paper reviews devices, emphasizing distinctions among representative apnea devices and technologies for home detection of OSA. The collected articles are analyzed to present a clear discussion. Each article is evaluated according to diagnostic elements, the implemented automation level, and the derived level of evidence and quality rating. The findings indicate that the critical variables for monitoring sleep behavior include oxygen saturation (oximetry), body position, respiratory effort, and respiratory flow. Also, the prevalent trend is the development of level IV devices, measuring one or two signals and supported by prediction software. Noteworthy methods showcasing optimal results involve neural networks, deep learning, and regression modeling, achieving an accuracy of approximately 99%.

## 1. Introduction

According to the American Academy of Sleep Medicine (AASM), sleep apnea is a severe disorder characterized by interrupted breathing during sleep [1]. Untreated sleep apnea in individuals involves frequent pauses in breathing, often happening numerous times throughout the night. If not addressed, this condition can result in loud snoring, daytime fatigue, and potentially more severe complications such as heart problems or high blood pressure [1]. The apnea–hypopnea index (AHI), which measures the frequency of respiratory events per hour [2], assesses the severity of sleep apnea. Despite the availability of various commercial devices for monitoring primary symptoms, it is necessary to make them more accessible to the general population. Additionally, sleep clinics often experience extended wait times. Consequently, there has been a surge in scientific research focusing on monitoring and predicting OSA. Worldwide, one in three individuals experiences a sleep disorder. In Mexico, for instance, 75% of sleep clinic patients suffer from snoring or sleep apnea, and 25% of them seek treatment for insomnia and other disorders [3]. Sleep apnea affects 4% of men and 2% of women in Mexico explicitly [4].

**Figure 1:**
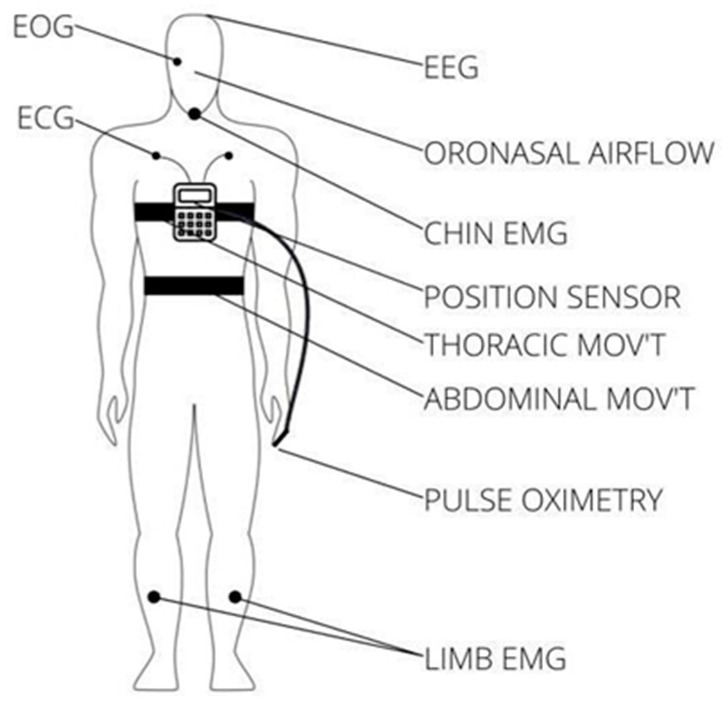
Position of each sensor in a PSG.

There are three main types of sleep apnea: OSA, central sleep apnea (CSA), and complex sleep apnea. OSA is the most common form when throat muscles relax. During these episodes, the diaphragm and chest muscles work harder than average to open the airways. The patient may start to breathe with loud gasps or jerk their body. Central sleep apnea occurs when the brain does not send a signal to breathing muscles properly. Instead, the brain fails to tell the muscles to breathe because of issues in the respiratory control center. It is related to the function of the central nervous system. Complex sleep apnea syndrome, or treatment-emergent central sleep apnea, occurs when someone has obstructive and central sleep apnea [1]. Sleep apnea is typically diagnosed through a standard test called nocturnal polysomnography (PSG) [5]. Monitoring equipment connects the patient and tracks nine specific variables during this test. The screening study can be conducted in a sleep laboratory or at home if specialized equipment is available. These variables include oximetry, respiratory flow, respiratory effort, actigraphy, electroencephalography (EEG), muscle electrical activity (EMG), eye movements, heart rate, and snoring [5]. For reference, Figure 1 illustrates the placement of each sensor on the human body in a PSG set-up [6].

Home sleep testing is also an option for detecting OSA [7]. Despite its effectiveness as the primary method for detecting sleep apnea, polysomnography frequently leads to prolonged diagnostic wait times due to the condition manifesting during nighttime sleep. Furthermore, sleep laboratories specializing in diagnosing sleep apnea may have limited capacity to evaluate all patients.

Alternative solutions for diagnosing OSA include portable home devices, such as portable monitors or out-of-center sleep tests that measure fewer variables [7]. One notable characteristic of these mobile devices is their cost-effectiveness compared with polysomnography. However, it is essential to note that according to the AASM, portable home devices are recommended only for patients with a high likelihood of having sleep apnea. One of the main limitations of these devices is the absence of an electroencephalography (EEG) monitor. Although this may not initially seem significant, an EEG monitor is valuable, as it helps determine the patient’s wakefulness and sleep stages, providing a more accurate assessment of the presence and severity of OSA. It is essential to note that if a patient is suspected of having OSA but receives a negative result from a portable home device test, they should undergo polysomnography for additional diagnostic evaluation. [7].

Some individuals may still need to be diagnosed when sleep laboratories require additional resources to analyze all patients. Furthermore, specific communities may perceive the available diagnostic systems for sleep apnea as relatively inexpensive. According to [8], 27.3% of adults in Mexico face an elevated risk of OSA, with a predominant concentration in urban areas and an average age of around 40 years old. As age increases, the likelihood of reporting insomnia, insufficient sleep duration, and a high risk of OSA increases. In Mexico, the prevalence of OSA has significantly risen from the original reports of 2% in women and 4% in men to 23% in women and 26% in men [8]. 

This paper presents various hardware and software technologies developed for sleep apnea detection. It aims to analyze the advantages and disadvantages of each technology and propose a solution that incorporates the most notable elements from the reviewed approaches. The paper comprises five sections: Methodology, Modules, Technologies for Apnea Detection, Results, and Conclusions. The methodology section establishes the critical variables to be discussed and compared. The third section summarizes commercial devices to monitor PSG signals, hardware and software solutions in scientific research papers, and regulations for medical devices regarding materials and sensors. This section is relevant as it presents information comparing approaches to the same issue: OSA diagnosis. The tables provide a quick review of the main characteristics of each solution. Finally, in the conclusion, we propose a solution with the main advantages based on the variables discussed earlier.

## 2. Methodology

This paper comprehensively studies commercial and independent devices and solutions to detect OSA in a period lapse of 10 years. Over the years, advancements in research have enhanced the detection of OSA, resulting in the phasing out of numerous devices either due to aging or the introduction of updated versions. Also, researchers have developed new detection algorithms, yielding better accuracy and specific results; thus, older articles are no longer an optimal choice.

This study used the Preferred Reporting Items for Systematic Reviews and Meta-Analyses (PRISMA) method [9] to conduct the review. The PRISMA methodology involves identification, screening, and data extraction and synthesis. By following these steps, the review ensures meticulous execution, emphasizing pinpointing all pertinent studies and consolidating the results so others can reproduce and duplicate them. The research questions for the review are as follows:

- In the last ten years, what commercial devices have had the most significant impact on detecting OSA in the market?
- In the last ten years, what methods have been used to detect OSA using a low quantity of signals?
- In the last ten years, which devices have been developed for signal monitoring to diagnose OSA?
- Based on the gathered information, what is the most effective signal and method for diagnosing OSA?

**Figure 2:**
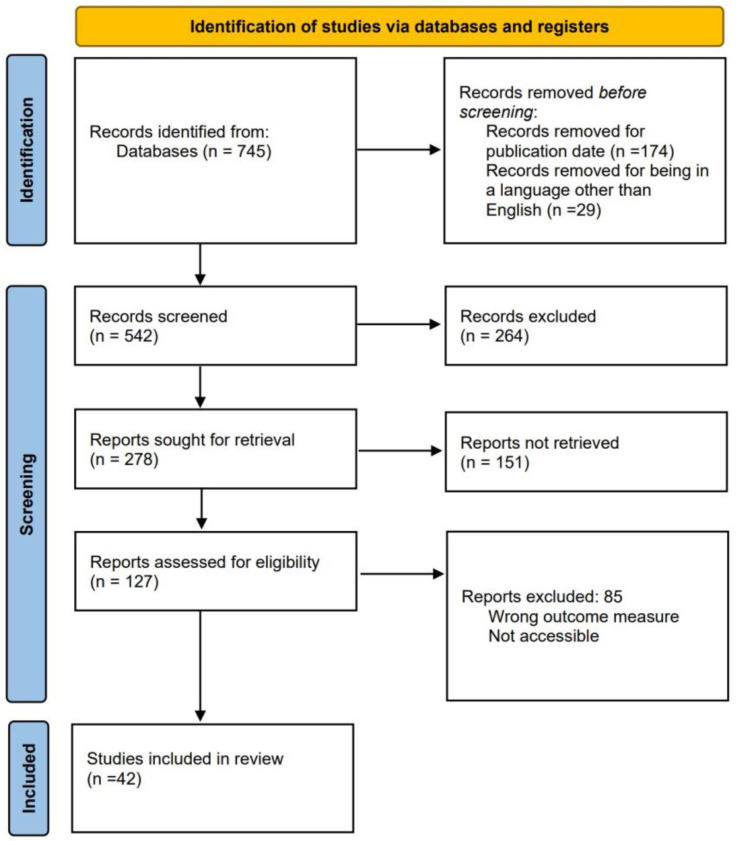
Methodology for the systematic review.

Figure 2 illustrates the methodology used to search and select records.

### Literature Review Process

Three primary searches were considered for the review. The first one is for selecting commercial devices capable of measuring variables that enable the diagnosis of OSA. The second search focuses on articles about applied algorithms for OSA detection. The third search is related to developed devices that monitor one or several signals used in PSG. The team conducted all searches on PubMed, a specialized database with numerous medical articles related to OSA. The publication timeframe was narrowed down to the past ten years, from 2013–2023, and the language was specified to only English articles. Keywords used for searching commercial devices were “OSA” AND “validation” AND “PSG”, with 298 results. Keywords used for the search of algorithm development were “OSA” AND “detection” AND “method”, with a total of 90 results. Keywords used for the search of independent hardware development were “OSA” AND “monitoring” AND “PSG”, with a total of 357 results. Figure 2 shows the process of reducing the total number of articles from 745 to 42. The reduction process employed three modules. In the initial module, 264 reports were excluded, and the title and abstract were scrutinized to eliminate those whose focus was unsuitable for the study. During the second module, 151 articles were discarded, and the results were analyzed to exclude those lacking any measurable value, specifically those that did not demonstrate accuracy, sensitivity, or specificity. In the third module, 85 articles were excluded, and a review of the methodology proposed by each report was conducted. Reports were discarded if the study’s data did not originate from a database or were collected without any criteria for selecting the chosen patients.

## 3. Modules and Technology Available for OSA Detection

This section is divided into four subsections: commercial devices, scientific research, regulation for recording modules, and regulations for materials. In the first two subsections, solutions for the monitoring and diagnosis of OSA are presented. The following two subsections introduce the regulations to develop an OSA monitoring device. It is crucial to summarize commercial and research proposals to discern the various solutions developed in recent years and delineate their respective strengths and weaknesses.

As will be seen in the following sections, commercial devices primarily monitor signals related to OSA. On the other hand, independently proposed methods not only concentrate on monitoring but also on diagnosing the disease using a fewer number of signals than commercial devices. By consolidating all the information gathered in this section, the characteristics that best fit new proposals for diagnosing OSA can be selected. The regulations section offers various rules to be followed when a medical device in this field aims to be distributed in specialized medical centers. The former adheres to medical standards, which must be followed for the product to be placed on the market and distributed to hospitals, given that this manuscript aims to summarize and identify the best solutions for OSA diagnosis.

### 3.1. Commercial Devices for OSA Diagnosis

According to the AASM, Portable Home Sleep Monitoring (PHSM) devices are classified into Types II, III, and IV [10]. It is important to note that portable Type III and IV monitors do not directly detect sleep stages. Instead, they estimate the respiratory disturbance index (RDI) or AHI by extrapolating data from the recorder’s active period [10]. Type II monitors have at least seven channels (e.g., EEG, EOG, electromyogram, heart rate, airflow, respiratory effort, oxygen saturation). This type of device monitors sleep staging and allows the calculation of AHI. Type III monitors are devices with a restricted number of channels, typically ranging from four to seven, and they must have at least four channels. The most common channels are heart rate, oxygen saturation, and respiratory measurements. Finally, Type IV devices were typically utilized to measure only one or two parameters, such as oxygen saturation or airflow, ultimately limiting their scope. However, this changed when the Centers for Medicare and Medicaid Services (CMS) decided to include coverage for continuous positive airway pressure (CPAP) treatment based on positive tests from Type IV devices with a minimum of three channels. Also, these devices can screen pediatric patients with OSA, as mentioned by [11].

In 2003, the AASM, the American College of Chest Physicians, and the American Thoracic Society recommended the diagnostic approach for patients suspected of having OSA; thus, they advised a complete polysomnography (PSG) study for patients with a strong suspicion. In cases where a portable monitoring (PM) device of Type II with a minimum of seven channels is utilized, it must be integrated into an attended PSG. Conversely, a four-channel study is conducted using a Type III portable monitoring (PM) device in a hospital setting supervised by a technician to assess the presence of OSA. However, an unattended four-channel study is not recommended. Lastly, an IV PM device with a single or double channel is also not recommended for diagnosing OSA [12]. These recommendations are related to the number of variables for each device, and it is important to note that all devices within these categories are intended exclusively for data collection, not diagnosis. Subsequently, a specialist reviews the collected information and, with additional signals from the patient, can formulate a more precise diagnosis of their condition. Nevertheless, new technology related to Type IV devices has been developed in recent years. Although this technology surpasses other methods for signal collection, it is not completely recommended, because it solely concentrates on monitoring a single signal, providing the specialist with limited information for an accurate diagnosis. Despite many recent OSA detection proposals with new technologies, PSG remains the gold standard.

In this section, several commercial devices are presented, selected from a 2018 review that showed 50 systems commercialized from 2000 to 2017 [13]. These devices were validated against PSG in the scientific literature and are described in that paper. Also, the devices were innovative in sleep medicine and have revolutionized the detection and management of sleep disorders. For instance, the Alice PDx by Philips [14] is a portable monitor for detecting sleep apnea and assessing cardiorespiratory disorders. This device was validated against PSG with a significant sample size of 85 patients, and it exhibited an impressive diagnostic agreement of 96.4%, as shown by [15]. An application related to aldosteronism and OSA detection using the Alice PDx can be found in [16]. The Apnealink Air by ResMed [17] is another home device that measures parameters such as respiratory effort, pulse, oxygen saturation, respiratory flow, and snoring. Its diagnostic capabilities encompass a range of diseases, including OSA. In the validation against PSG conducted in [18], Apnealink Air exhibited promising sensitivity at 94% and specificity at 29%, with improvement noted when the AHI threshold was adjusted to one hour. In addition, Apnealink Air was used in a study in the Hospital Sleep Unit (HSU) to compare its performance against polygraphy (RP) [19]. Another device is the WatchPat One [20], which has emerged as a revolutionary Home Sleep Apnea Device (HSAT), as it measures up to seven channels, providing more comprehensive data. Also, it includes a swift data analysis capability for identifying apnea events in just one minute. WatchPat One was validated against PSG in [21], and it was found to have a sensitivity of 95.8% and a specificity of 55%. Also, WatchPAT can determine OSA in patients with chronic obstructive pulmonary disease, as shown by the study conducted in 2022 to detect OSA in patients with Down syndrome [22]. 

Further, Embletta MPR [23] is a fourth-generation ambulatory recorder that offers the capacity to record data from seven channels, encompassing variables such as abdominal strain, chest strain, nasal pressure, nasal flow, snore, SpO2, heart rate, position, and audio. Crucially, this device permits the incorporation of additional sensors for a more comprehensive study, offering increased versatility in monitoring. Embletta underwent validation against PSG in [24], demonstrating a remarkable correlation in the AHI compared with PSG, boasting a sensitivity of 0.924 and a specificity of 0.857. Furthermore, a research study was conducted using the same device to predict severe OSA in patients [25]. Oppositely, the ARES Unicorder [26] takes a different approach, focusing on the user’s comfort, and it was designed for home-based sleep monitoring. Worn on the forehead, this device can measure actigraphy, pulse, oximetry, position, respiratory effort, nasal pressure, and audio for up to three nights for extended monitoring. In [27], the ARES Unicorder’s diagnostic sensitivity of 95% and specificity of 94% during in-lab testing for sleep-disordered breathing (SDB) was demonstrated using a respiratory disturbance index (RDI) cut-off of 15 per hour. Even when employed in a home setting, ARES Unicorder maintained a strong performance, with a sensitivity of 85% and specificity of 91%. Also, the ARES Unicode device was tested in a study showing the relationship between chronic rhinosinusitis and OSA [28].

Additionally, the Apnomonitor 5 can measure up to five signals: oximetry, position, respiratory effort, and audio. It was validated against polysomnography (PSG), which displayed a sensitivity of 95% in identifying moderate to severe sleep apnea cases [29]. This device was used to determine the relationship between sleep quality and sleep apnea [30]. Moreover, the Lifeshirt is an ambulatory monitoring device for multiple physiological parameters, including respiration, electrocardiogram activity (ECG), and the patient’s posture. Its versatility can be extended if additional sensors include pulse oximetry, EEG/EOG, temperature, blood pressure, and capnometry. The device’s validation against PSG [31] reinforced its effectiveness, with sensitivity ranging from 0.85 (for AHI = 5) to a perfect 1.00 (for AHI = 25) and specificity ranging from 0.67 to 1.00. By utilizing the Bland–Altman technique for determining agreement, the mean difference between the AHI recorded by the Life shirt and PSG was 1.02, with a margin of error of ±16.36. Also, the Lifeshirt was used in the study presented by [32] to evaluate the possibility of measuring inhalation patterns. Another device is the SOMNOcheck micro [33], which was designed for sleep and cardiovascular studies by monitoring pulse, oximetry, body position, nasal pressure, and audio. It can validate the presence of obstructive or central apnea events and provides essential oxygen desaturation statistics. The device’s validation against PSG further establishes its credibility and reliability as a critical tool in sleep medicine and the assessment of cardiovascular health, as presented by [34]. Additionally, this device was used in a 2018 study to evaluate sleep questionnaires [35].

MediByte is another commercial device [36], and it is described as the world’s smallest recorder, measuring a mere 2.5 × 2.25 × 0.75 inches (66 × 60 × 19 mm) and weighing 3.3 ounces (93 g). It offers compatibility with CPAP devices through the Luer connector. This device measures ECG, oximetry, effort, and nasal pressure. In a thorough validation against PSG, as detailed in [37], MediByte achieved a sensitivity of 80% and a specificity of 97%, which is especially noteworthy when considering an AHI threshold exceeding 15 events per hour. Moreover, with a higher threshold (AHI exceeding 30 events per hour), the device demonstrated a positive predictive value of 100% and a negative predictive value of 88%. An essential application of this device in research involving pediatric patients against PSG is described in [38]. Meanwhile, ApneaLink Plus [39] offers a convenient and effective solution for home-based sleep studies categorized as Type III diagnostic equipment. This device empowers patients to undergo sleep assessments in their homes, recording parameters such as respiratory effort, pulse, oxygen saturation, and nasal flow. It reports sleep events, including apneas, hypopneas, flow limitation, snoring, blood oxygen saturation, and the likelihood of Cheyne–Stokes respiration (CSR). ApneaLink Plus demonstrated its reliability, boasting a specificity of 93% in a meticulously validated study against PSG with many subjects [40]. Additionally, this device was employed in a recent study [41] to delineate objective measures of post-intracerebral hemorrhage and sleep-disordered breathing.

The NOX T3 [42] is another sleep monitoring device designed to cater to adults and pediatric patients. It includes Bluetooth pulse technology, a cutting-edge innovation developed by Nox Medical, and collects data to be analyzed by its software. Its performance was assessed against PSG in [43], considering an AHI threshold of five events per hour. At this threshold, it attains a sensitivity of 95% and a specificity of 69%, with a positive predictive value of 94% and a negative predictive value of 75%. Setting the AHI threshold at 15 events per hour, the device retained a sensitivity of 93%, a specificity of 85%, a positive predictive value of 89%, and a negative predictive value of 91%. Besides, another study used the NOX T3 to evaluate its accuracy against random tests [44].

Table 1 compares the described devices whose validation or application date is from 2018 to 2022. The validation column shows articles where the device accuracy was obtained. The application column shows articles where the device was used to detect OSA. The exclusion of Type IV devices from the table occurred as they primarily rely on software solutions, a topic discussed in the software section of this paper.

While a clinical trial remains the best method to validate an HSAT, other alternative approaches exist. Two strategies without machine learning are employed for developing automated scoring algorithms. The first strategy involves a validation cohort of approximately 30–100 subjects who undergo simultaneous testing with a gold standard. The device utilizes its features to predict an output label, such as sleep stages, as defined by the gold standard. The second strategy is to transfer validation, which compares the results with a large dataset.

Another approach to validate an HSAT is using clinical guidelines. In 2007, the AASM published the first clinical guideline for using limited channel kits for sleep apnea diagnosis, which was updated in 2017. The “Clinical Practice Guideline for Diagnostic Testing for Adult OSA: An AASM Clinical Practice Guideline” recommends diagnosing OSA in adults. These recommendations guide clinicians categorized as STRONG or WEAK according to their certainty level and appropriateness for patient care under the GRADE methodology [1].

### 3.2. Hardware and Software Systems for OSA Diagnosis Available in Scientific Research

Unlike commercial devices, scientific research proposals have been focused on developing software for predicting OSA based on a few input sensors and machine learning algorithms. Also, many solutions have been proposed through alternative hardware, such as smartwatches and rings [45]. Moreover, according to [46], between 2016 and 2019, numerous Internet of Things (IoT)-based solutions emerged; thus, collaborative efforts in this domain, leveraging technologies like smart devices, fog computing, cloud, big data, and machine learning, have facilitated the development of innovative solutions. According to [47], the proliferation of wearable watches with photoplethysmography (PPG) sensors allows the monitoring of continuous pulse wave data during daily activities. This study investigated the use of PPG data from a smartwatch for diagnosing OSA, showing that smartwatch information can be a viable alternative with a final accuracy of 85%. Another example of a smartwatch application is shown in [48]. This study uses a smartwatch and a smartphone to record body signals: the smartwatch’s accelerometer and heart rate monitor are used together with the sound level sensor of a smartphone. Even though these devices are not particular for OSA monitoring, the application exemplifies how new technologies are used. An example of a finger ring used to diagnose OSA is the Belun Ring Platform [49]. This device is a scientific research proposal and can measure oxygen saturation, photoplethysmography, and accelerometer signals. It showed a sensitivity and specificity of 0.85 and 0.87, respectively. 

Other recent trends include the development of smartphone apps, as highlighted in [50]. While these apps may have a promising future, they are less accurate than traditional methods. An example is introduced by [51], where a wireless pulse oximeter is used together with an app to diagnose OSA. In this solution, the smartphone did not record information from any internal sensor but instead received data from the oximeter. Further, [52] demonstrates that app-based solutions are unreliable, requiring an expert review for an accurate diagnosis. The primary benefits of these solutions include their noninvasiveness and the avoidance of patients needing to visit a sleep center for a comprehensive diagnosis. Nevertheless, since preventive studies do not encompass the monitoring of all variables measured in a PSG study, the diagnosis should be reassessed by a sleep expert. As reported in [53], recent years have witnessed the emergence of novel detection methods based on noncontact sensors, such as radio frequency, audio, and video. In [54], a proposal for a video-based OSA diagnosis system is presented, comparing PSG with SleepWise, which is a noninvasive technology relying on image processing. Sleepwise demonstrated a sensitivity of 100% and a specificity of 83%. Another method for OSA detection involves sound levels. As outlined in [55], a proposal for diagnosing OSA based on sound and deep learning achieved a final sensitivity of 95.6% and specificity of 91.6%. Another sound-based solution is presented in [56], incorporating tracheal breathing sounds alongside pulse oximetry records. The study demonstrated sensitivity and specificity values exceeding 91%. Furthermore, radiofrequency has been suggested as an unconventional signal for diagnosing this condition, such as the solution described in [57], which was introduced based on impulse-radio ultra-wideband radar, attaining a final agreement of 0.93 for the model. 

In [58], an engineered wearable home system is introduced for prescreening and ongoing monitoring of sleep-related breathing disorders. This system, positioned on the nasal septum, includes critical components like photoplethysmography, an accelerometer, a microcontroller, and a Bluetooth transmission unit. It identifies apnea episodes by analyzing the photoplethysmography signal modulation during the respiratory cycle. The accelerometer distinguishes between obstructive and central apnea types by detecting thoracoabdominal movements. This system achieves exceptional sensitivity and precision, around 90%, detecting and monitoring over 500 apnea episodes. This development signifies a promising advancement in sleep-related disorder diagnosis and at-home monitoring.

In [59], a study is presented with the primary objective of evaluating the potential of a microbend fiber optic sensor (MFOS) to detect vital signs and sleep apnea in the controlled environment of an in-lab sleep study. Ten participants underwent full polysomnography (PSG) with discreet placement of the microbend fiber optic sensor (MFOS) beneath the patient’s mattress to capture bed-embedded ballistocardiogram (BCG) data. In addition, the vital signs were assessed within a 30 s time frame with a 15 s overlap. Electrocardiograms and thoracic effort signals were critical reference points in the assessment process. The research outcomes revealed commendable results for sleep apnea detection, with an accuracy rate of 49.96%, a sensitivity rate of 57.07%, and a specificity rate of 45.26%. These findings suggest promising advancements in sleep-related disorder diagnosis in clinical settings using a nonintrusive and practical approach. A similar approach is presented by [60], where an autonomous system is introduced to detect sleep apnea signals using pressure sensors beneath the mattress. The system’s hardware includes several components, such as a Raspberry Pi, an analog/digital converter, an amplifier, a filtering circuit, and a force-sensitive resistor. The system captures the patient’s breathing signal and is transmitted to the Raspberry Pi at a voltage level. There, the software is crucial for filtering and amplifying the signal for a more precise analysis. In the end, the system achieved an overall accuracy rate of 91% in recognizing apnea occurrences, showcasing robust performance. It also maintained an average recognition delay of approximately 15 s, highlighting its potential for future sleep apnea detection and monitoring applications. An alternative noninvasive device is suggested in [61]: an automatic scoring algorithm examines the blood oxygen saturation signal on a minute-by-minute basis. Statistical and frequency-based features are extracted and input into a classifier. The ratio of the time of OSA episodes to the total time in bed is compared with a threshold for a comprehensive OSA diagnosis. The device attained an accuracy of 88%, a sensitivity of 80%, and a specificity of 91%. 

Another study with an innovative monitoring system is proposed in [62], using a tracheal sound (TS) sensor during sleep to identify apnea. Polysomnographic recordings from 32 patients served as the dataset, enabling an efficacy comparison of four airflow signal methods: the oronasal thermal airflow sensor (thermistor), a nasal pressure transducer (NP), respiratory inductance plethysmography (RIPsum), and the TS. Notably, the thermistor signal served as the reference for scoring, and it showed that with this method, there were 4167 apneas detected: 5416 with the NP, 2959 with the RIPsim, and 5019 with the TS caught. The findings suggest that placing TS sensors has the potential to identify apneas that might be overlooked by RIPsum and detect apneas that NP sensors may miss. However, it is essential to note that TS sensors may tend to overscore apneas due to mouth breathing.

Other studies involve recent technology trends; for instance, ref. [63] incorporated IoT to introduce an innovative, wearable, and energy-efficient system designed for long-term monitoring of OSA. The system’s advantage lies in its embedded IoT infrastructure, connecting home health care with professional supervision. It utilizes a single-channel ECG for monitoring the patient, employing silver chloride electrodes for impedance-pneumography-based ECG measurement. A noise filter is applied to eliminate artifacts, and an ECG delineation process extracts key fiducial points using wavelet transforms. The results validate two automatic diagnostics: one for detecting OSA and the other for monitoring the patient’s cardiac status. This system demonstrates a classification accuracy of up to 88.2%, highlighting its potential as an accurate and practical tool for long-term OSA monitoring. Table 2 summarizes the different hardware-based references, the number and type of sensors, and their observed performance. 

Also, there are scientific research papers based on software development for predicting OSA. An example of a noncontact method for estimating OSA is provided in [64]. The main objective of this study is to pioneer a noncontact method aimed at assessing the severity of sleep apnea while discerning between positional and nonpositional sleep apnea cases. This cutting-edge approach leverages the power of a deep learning algorithm, which diligently scrutinizes infrared sleep videos to gauge and quantify the AHI. A noteworthy facet of this algorithm is its capacity to pinpoint patients affected by positional sleep apnea, a condition predominantly associated with individuals who favor sleeping on their backs [65]. The core of this innovative system is a 3D convolutional neural network (CNN) architecture, providing robust analytical capabilities. The algorithm identifies positional sleep apnea patients by combining information from AHI with data on sleeping positions, achieving an accuracy rate of 83% and an F1 score of 86%. The effectiveness of this method is substantiated through validation with data from a cohort of 41 participants (26 men and 15 women), highlighting its potential to advance sleep apnea diagnostics and provide insights into positional sleep apnea identification.

In pursuit of innovative sleep apnea detection methods, ref. [66] presented a novel approach for contactless and automated sleep apnea detection by analyzing snoring signals. Leveraging the power of hybrid deep neural networks, the researchers collected a substantial dataset of 5700 snoring segments from 32 patients. They further enhanced the analysis by extracting Mel filter banks (FBanks) [67] from the snoring sounds, which provided a superior resolution at lower frequencies while reducing resolution at higher frequencies. A classification model was constructed employing this enriched dataset, harnessing the capabilities of hybrid deep neural networks. The system achieved an average classification accuracy of 74.27% across categories, spanning obstructive apnea, central apnea, hypopnea, and regular snores. This methodology marks significant progress in sleep apnea diagnosis, offering a contactless and automated solution with high accuracy and potential for clinical application. Additionally, an innovative study proposed by [68] introduces a multiscale neural network named SE-MSCNN, which plays a central role in detecting sleep apnea (SA) by utilizing single-lead ECG signals. The authors conducted comprehensive experiments using the PhysioNet Apnea-ECG dataset to showcase the remarkable performance of SE-MSCNN. This neural network achieved results with the highest per-segment performance metrics, including an impressive sensitivity of 86.00%, a specificity of 93.52%, and an accuracy of 90.64%. Furthermore, the system exhibited unparalleled per-recording performance, achieving a perfect score with 100% sensitivity, 100% specificity, and 100% accuracy. These outcomes signify an important advancement in sleep apnea detection, providing a lightweight yet effective solution with immense clinical potential.

Further, a novel approach is introduced in [69], presenting an automatic feature extraction method that combines convolutional neural networks (CNNs) and long short-term memory (LSTM) recurrent networks to accurately differentiate individuals with apnea from those without, employing the apnea–hypopnea index (AHI) as a crucial diagnostic measure. The method demonstrates advancements, featuring a sensitivity of 94.41%, a specificity of 98.94%, and an overall accuracy of 97.21%. Furthermore, extensive testing on the St. Vincent’s University Hospital/University College Dublin Sleep Apnea Database (UCDDB) dataset underscores its robustness, achieving a high accuracy rate of 93.70%, sensitivity of 90.69%, and specificity of 95.82%. A deep learning approach establishes the credibility of deep learning methodologies in diagnosing OSA, utilizing electrocardiogram (ECG) signals as the primary diagnostic modality [70]. The ECG signal undergoes meticulous preprocessing, normalization, and segmentation into 10 s intervals for efficient analysis. With data from 86 patients, the study allocates 69 patients’ data for training. It reserves the remaining 17 patients’ data for testing. The best-performing model achieves an exceptional accuracy rate of 99%, emphasizing the potential of deep learning to enhance the accuracy and effectiveness of OSA diagnosis. In [71], a decision support system is introduced to identify individuals with OSA. Diverging from other methodologies relying on multiple parameters from polysomnographic data, this system exclusively focuses on the Pulse Transition Time (PTT) parameter. Deep learning techniques are used to extract pertinent features from PTT signals. The study utilizes two convolutional neural network (CNN) models, AlexNet and VGG-16, for feature extraction. Comparative evaluations with other studies in the existing literature affirm this approach’s commendable performance and efficacy in OSA identification. Furthermore, the SpO2 signal is used as an input for an automated sleep apnea screening method [72]. This method identifies apnea and hypopnea events within the blood oxygen saturation (SpO2) signal. The study employs the six most discriminative features to develop classifiers for predicting whether respiratory events lead to desaturations. Furthermore, among the used classifiers, the random forest classifier stands out. This metaestimator utilizes multiple decision tree classifiers on various dataset subsamples, enhancing predictive accuracy through averaging [73]. The criterion for determining the presence of Sleep Apnea–Hypopnea Syndrome (SAHS) revolves around the number of desaturations per hour. The different test sets demonstrate an average classification accuracy of 82.8% in desaturation detection. Additionally, individuals with SAHS having an AHI exceeding 15 can be accurately identified with an average accuracy rate of 87.6%. One of the features sought when developing a solution for apnea prediction is to reduce the diagnosis time. In this context, [74] proposes a rapid and portable method to detect sleep apnea. The study introduces an approach that employs a neural network within a defined time window for detecting sleep apnea, focusing on a single-lead ECG signal. The PhysioNet Apnea-ECG [75] dataset is leveraged for investigation. Notably, the choice of time window size significantly influences the detection method’s performance, leading to the exploration of 16 different window sizes from 0 to 16. To evaluate the model’s effectiveness, the dataset of 70 recordings is partitioned into seven segments, with six segments assigned for training and the remaining portion reserved for testing in each partition. The study’s outcomes reveal that the optimal accuracy, reaching 87.3%, is achieved with a time window size of 10, emphasizing the significance of this approach for rapid and effective sleep apnea detection.

The study in [76] introduces an innovative approach to real-time sleep detection, utilizing convolutional neural networks (CNNs) and a single-channel nasal pressure signal. The research analyzes 179 polysomnographic recordings as its dataset. Nasal pressure signals undergo adaptive normalization and segmentation using a sliding window technique for analysis. The investigation reveals performance metrics with a sensitivity of 81.1%, a specificity of 98.5%, and an overall accuracy of 96.6%. This novel method is promising for accurate and real-time sleep detection applications. Another real-time application for OSA is presented in [77]: the central goal of this study is to propose an innovative method for near real-time automatic detection of apneic events. Leveraging a dataset of 230 polysomnography (PSG) records, including apneic events ranging from 0 to 86.5 events per hour, the research team conducted a thorough analysis. By scrutinizing quantitative features related to fluctuations in blood oxygen saturation attributed to apneic events, a set of criteria was meticulously established to identify such events reliably. The outcomes of this endeavor showcased a commendable achievement, with the model delivering an average accuracy rate of 96.7%. This new approach exhibits significant promise in advancing the real-time detection of apneic events in a clinical setting. 

More signals for OSA diagnosis have been explored in scientific research. For instance, [78] proposes a method based on mandibular jaw movements (MJM). A total of 67 patients with OSA were included in the study, where simultaneous PSG and MJM recordings were conducted. The model exhibited excellent agreement on an epoch-by-epoch basis (Kappa = 0.799), and a balanced accuracy of 86.6% was achieved for detecting MJM events based on RMMA standards. ECG stands out as one of the most common variables for OSA prediction. Similarly, [79] proposes a new deep-learning model based on the ECG signal from patients. A hardware solution was developed to validate the algorithm, and the ECG sensor was connected to a smartphone in which the method was implemented. A final accuracy of 92.15% was achieved.

Finally, two of the most recent approaches from 2023 are described in [80,81]. The work in [80] introduces a pioneering solution for OSA diagnosis using EEG signals. The article presents an innovative EEG Multi-Instance Learning Network (EEG-MIL) framework. The EEG-MIL has two key components: the Subframe Multi-Resolution Convolution Extractor (S-MRCNN) and the Multi-Instance Learning (MIL) mapping function. When functioning in tandem, these components extract essential features from subframes and unveil intricate relationships among various instances (sub-frames) and bags (frames). Notably, this model outperforms existing methods, showcasing a significant performance enhancement ranging from 2% to 8.6%. Consequently, the EEG-MIL framework establishes itself as the new state-of-the-art approach in OSA diagnosis, mainly using EEG signals. This advancement represents a noteworthy contribution to improving the accuracy and effectiveness of OSA diagnosis. Furthermore, [81] introduces a new OSA detection approach based on a single-channel EEG signal. The EEG Collaborative Learning Network (EEG-CLNet) algorithm is designed to perform concurrent sleep staging and OSA event detection. This method treats various tasks as a unified entity to extract features within the same groups by employing local parameter sharing and Cross-Task Knowledge Distillation (CTKD). The experimental outcomes indicate that the approach yields a performance improvement ranging from 1% to 5% compared with the baseline. The EEG-CLNet can reduce the overall number of model parameters and improve the model’s functionality. Table 3 shows the reviewed articles and their inputs, methods, and observed accuracies. It is essential to mention that the accuracies cannot be compared since each method compared its results with different baselines like PSG or a database. Thus, a direct comparison is not possible under these conditions. 

### 3.3. Applicable Regulations for Signal Monitoring Modules

Oximetry: Pulse oximeters employ the principle of differential light absorption to determine SpO2. These devices utilize a sensor placed on a body region, such as a finger, toe, or earlobe, to transmit light of different wavelengths through the skin. The primary standard applicable is ISO 80601-2-61 [82]. The Pan American Health Organization and the World Health Organization developed the Technical and Regulatory Aspects of the Use of Pulse Oximeters [83], where some of the following specifications are mentioned: SpO2 detection to include the range 70–99%, SpO2 resolution of 1% or less, SpO2 Accuracy (in the range at least 70–99%) within ± 3%, pulse rate detection range to include 30–240 bpm, and others.

Respiratory flow: The primary standard that applies, in this case, is ISO 23747:2015, titled “Aesthetic and respiratory equipment—Peak expiratory flow meters for assessing pulmonary function in spontaneously breathing humans” [84].

Respiratory effort: The standard applicable is ISO 4135:2022 Anaesthetic and respiratory equipment [85]. This ISO standard ensures the uniformity of terminology used in all relevant anesthesiology and respiratory care equipment standards. It establishes a common language that manufacturers, test laboratories, and regulatory agencies can use to effectively communicate and regulate such equipment.

Actigraphy: Although no principal applicable standard exists for an actigraph, the ISO 13485:2016 Medical Devices—Quality Management Systems—Requirements for regulatory purposes can be used [86]. According to the company Actigraph, which develops Medical-Grade Actigraphy monitors, their devices are certified by ISO 13485:2016, the European Union Medical Device Directive (EU MDD) 93/42/EEC, the Health Canada Medical Devices Regulations (CMDR), and the US FDA’s Quality System Regulations (QSRs) [87].

### 3.4. Applicable Regulations for Medical Materials

ISO 10993-1 identifies the standard endpoints for evaluating the biological effects of skin contact devices, which include cytotoxicity, sensitization, and irritation [88]. The FDA considers these factors when guiding the biocompatibility of specific devices in contact with intact skin. Further, this guidance only applies to medical devices composed of certain materials, including synthetic polymers, polycarbonate, polyoxymethylene, and some fabrics, including Lycra.

## 4. Results

Over the years, there has been a notable increase in commercial devices monitoring signals related to OSA, paralleled by a growth in publications addressing OSA detection. According to [89], OSA-related publications have shown an annual increase, with the United States leading in publications over the past decade. China has also seen a rise in OSA-related publications. A total of 24,291 OSA-related articles were reviewed from 2011 to 2020. Despite the surge in publications on sleep apnea, there is a demand for higher-quality research and the development of improved detection and treatment methods.

Mobile applications have become a trending technology in the field, with many developers using them for OSA detection. The emergence of “on-a-chip” and “smartphone” technologies has facilitated the rise of affordable at-home applications and devices focused on sleep health and disorders [50]. However, it is essential to note that while ten smartphone apps are available for diagnosing OSA, their accuracy is less reliable than traditional options. Further validation and testing in specialized centers are necessary for these apps to be considered accurate and reliable.

As outlined in the section on apnea detection modules and technology, diverse devices are accessible for monitoring various variables associated with OSA. Table 1 emphasizes Type III devices, which measure fewer variables, resulting in reduced production costs. The critical variables include oxygenation, respiratory flow, respiratory effort, ECG, and body position. Notably, there is a demand for more commercial Type IV devices, measuring only one or two variables as per AASM recommendations, owing to their limited accuracy. Many devices feature a modular design, comprising a central device or module to which variable-specific modules can be added based on the patient’s needs. Examples of such devices include Apnea Link Air, ApneaLink Plus, Alice PDx, and the ARES Unicorder. Accessing commercial devices for OSA detection can be challenging, often necessitating involvement from healthcare centers.

In scientific development, hardware devices strive to be nonintrusive, portable, and suitable for home studies without constant medical supervision. The aim is to miniaturize sensor modules to ensure compactness and ease of transportation. Wireless technologies, such as Bluetooth or Wi-Fi, are commonly employed for data communication. The primary variables in scientific hardware development are oxygenation and ECG, with a secondary objective of reducing energy consumption to enable extended monitoring periods of up to 12 h.

Scientific software development in the field has exhibited consistent characteristics over the past five years. Unlike commercial devices, Table 3 illustrates that all software development papers monitor a single variable (Type IV devices). The ECG signal is the most frequently utilized variable in research due to its rich information content. The commonly employed technologies are deep learning, neural networks, artificial intelligence, and regression models. Deep learning has shown superior performance among these methods, reaching accuracy levels of up to 99%. When coupled with the ECG signal, neural networks achieve an accuracy of 97%. Despite certain studies suggesting new variables, such as patient movements during sleep or infrared video signals for position detection, the processing of these variables has not yielded favorable results.

ECG is the predominant variable in hardware devices. Meanwhile, machine learning has shown promising results in software development. Among the mentioned articles, [66] combines both elements and achieves the highest accuracy (99%). The study proposes six machine learning solutions for sleep apnea prediction from ECG signals, including deep neural networks (DNN), one-dimensional (1D) convolutional neural networks (CNNs), two-dimensional (2D) CNNs, recurrent neural networks (RNNs), long short-term memory (LSTM), and gated-recurrent unit (GRU). The GRU model and CNN demonstrate the best accuracy levels. GRU, a simplified version of LSTM, is a recurrent neural network capable of learning sequence patterns without memory units. The 1D CNN model, utilizing ECG signal amplitude (V) as input, outperforms the 2D CNN model, achieving an accuracy level of 98.5% compared with 95.9% [71].

In the methodology section, the crucial variables included EEG, EOG, electromyogram, heart rate, airflow, respiratory effort, oxygen saturation, and ECG. To finalize the variable and method selection, an analysis of articles and solutions focusing on each variable was conducted, considering both the presence of the variable and the achieved accuracy. The choice of processing and variable was determined by prioritizing importance. Notably, snoring was identified as the least significant variable due to its infrequent inclusion in compared devices and the challenges associated with accurate processing for a proper diagnosis. Only one reviewed article utilized snoring, and its accuracy using this signal was 74.27%.

## 5. Discussion

Table 1 presents a comparison of the previously discussed commercial devices. Their primary distinction lies in the number of signals they can monitor, leading to the categorization mentioned. Despite differences, these commercial devices share a crucial similarity in the signals they monitor, aligning with those recorded in a PSG study (oximetry, respiratory flow, respiratory effort, actigraphy, EEG, EMG, eye movements, heart rate, and snoring [5]). These devices are typically utilized in specialized study centers like sleep clinics, limiting public access. Their high cost, approximately USD 500, is attributed to the quality of the sensors. Additionally, comparisons against PSG studies are essential for validating their reliability, prompting articles to conduct such assessments.

Some devices, such as WatchPat One [16], differ by providing specific annotations on signals, including sleep cycle identification and anomalies, aiming to facilitate specialist diagnosis. However, it is crucial to note that none of these devices are designed for OSA diagnosis; their primary role is signal monitoring. While their results contribute to a diagnosis in specialized centers, they do not conclusively establish whether a patient has sleep apnea.

Scientific research devices can be categorized into those developing hardware solutions for signal monitoring and those focusing on applying algorithms for OSA detection. Like commercial devices, hardware solutions are segmented based on the number and type of sensors. While some references propose innovative solutions with sensors beneath the mattress [60], most devices measure traditional PSG variables. Despite measuring the same signals, each solution employs different types of sensors. Blood oxygenation (measured with a finger sensor) and ECG (with electrodes on the chest) are the two most frequently used signals among hardware solutions.

Some solutions focus on applying algorithms for OSA detection, as shown in Table 3. Among these, five solutions utilize ECG signals as input for their algorithms. Notably, [71] achieves the highest accuracy, employing the ECG signal in combination with deep learning to achieve an accuracy of 99%. Consequently, it can be inferred that the ECG signal is frequently utilized in independent methods due to the wealth of information that can be extracted through processing. The most effective methods identified in the review include deep learning, deep neural networks, regression modeling, and convolutional neural networks.

## 6. Conclusions

OSA has garnered substantial attention, resulting in increased efforts for independent and commercial developments to tackle this issue. Commercially available devices mainly belong to Type III, emphasizing ECG and oximetry variables. On the scientific front, single-variable approaches, enhanced by postprocessing techniques, offer reasonably accurate predictions. Notably, artificial neural networks and deep learning methods have emerged as prominent strategies for postprocessing, achieving accuracy rates of up to 99% when combined with the ECG variable.

Based on the analysis of existing research, a new system for detecting sleep apnea can be proposed, integrating the best-performing characteristics identified in current studies. However, the systems discussed in the articles face a significant challenge: their dependence on wireless communication between central and external modules. It is crucial to acknowledge that medical regulations overseeing wireless devices can be intricate due to the susceptibility of wireless communication to external noise interference.

A future direction involves the development of a device specifically designed to predict sleep apnea in Mexican patients. This device should leverage the best-detected characteristics while prioritizing cost-effectiveness. It is essential to weigh the advantages and disadvantages of commercial and independently developed devices, emphasizing the need to define the primary objective and approach of the proposed device. A novel and effective sleep apnea detection system could be achieved by carefully considering these factors.

## Figures and Tables

**Table 1:** Comparison between commercial devices for OSA detection validated in the scientific literature.

Device	Measurements	Category	Validation	Application
Alice PDx [14]	Oxygen, SpO2, Heart Rate, Snoring, Respiratory Flow	III	Eighty-five patients with suspected OSA were studied. The Alice PDX was used in diagnostic agreement with PSG in 96.4% of the studies [15].	The study was conducted involving subjects from Indonesia. Its main objective is to prove that there is a correlation between primary aldosteronism and OSA [16].
Apnea Link Air [17]	Oxygen, SpO2, Heart Rate, Snoring, Respiratory Flow	III	Sixty children and adolescents with suspected OSA were studied. When the AHI threshold was adjusted to 1 h, the sensitivity and specificity were 94% and 29% [18].	Comparison between the interpretation of Apnea link Air by primary care physicians (PCPs) and the one achieved through respiratory polygraphy (RP) at the Hospital Sleep Unit (HSU) [19].
WatchPat One [20]	PAT signal, Heart Rate, Oximetry, Actigraphy, Body, Position, Snoring, Chest Motion	II	A total of thirty-six subjects exhibiting suspected OSA underwent an examination. The obtained sensitivity of WatchPAT One at an AHI cut-off of ≥5 was 95.8% [21].	To detect OSA in patients with Down syndrome using the WatchPat One. OSA was seen in 95% of participants [22].
Embletta [23]	Abdominal Strain, Chest Strain, Nasal Pressure, Nasal Flow, Snore, SpO2, Heart Rate, Position, Microphone	II	Eighty subjects exhibiting suspected OSA underwent examination. The sensitivity at an AHI > or =5 h was 0.924 [24].	The Embletta device was used in a study to predict severe OSA in patients awaiting sleep studies [25].
ARES Unicode [26]	Actigraphy, Pulse, Oximetry, Position, Effort, Nasal Pressure, Audio	II	The study was conducted involving eighty subjects with suspected OSA and twenty-two volunteers. The sensitivity and specificity of ARES Unicode were 85% and 91%, respectively [27].	A study to examine the relationship between chronic rhinosinusitis and the prevalence and severity of OSA [28].
Apnomonitor 5	Oximetry, Position, Respiratory, Audio, Effort	III	Twenty-two adults with suspected OSA underwent an examination. The sensitivity of the Apnomonitor 5 was 95% against PSG [29].	The study aimed to identify the relationship between quantitative measures of sleep quality, sleep apnea, autonomic function, and insulin secretion and sensitivity [30].
Lifeshirt	EOG, Pulse, Oximetry, Respiratory Flow	III	Fifty subjects with suspected OSA underwent examination. The sensitivity of the Lifeshirt ranged from 0.85 to 1, depending on the AHI. Specificity ranged from 0.67 to 1.00 [31].	The application aimed to examine the possibility of modifying Wireless Respiratory Monitors (WRMs), like the Lifeshirt, to measure inhalation patterns [32].
SOMNOcheck micro [33]	Pulse, Oximetry, Position, Nasal pressure, Audio	III	The study involved one hundred five subjects with suspected OSA. There were no differences between the AHI of the device and PSG [34].	The study aimed to assess the effectiveness of various sleep questionnaires. Subsequently, PSG and other evaluation methods were conducted, and the questionnaires were administered again after treatment [35].
Medibyte [36]	ECG, Oximetry, Effort, Nasal Pressure	III	Seventy-three subjects with suspected OSA were involved in the study. The sensitivity and specificity of the screener were 80% and 97%, respectively [37].	The study aimed to validate the Medibyte device by comparing it with PSG in pediatric patients who wore both setups simultaneously [38].
ApneaLink Plus [39]	Respiratory effort, Pulse, Oxygen saturation, Nasal Flow	III	The study involved one hundred fifty subjects with suspected OSA. The specificity of the device was 93% [40].	A study aimed to characterize the objective measures of sleep-disordered breathing (SDB) in patients with post-intracerebral hemorrhage. The Apnea Link Plus was used to screen SDB [41].
NOX T3 [42]	Oximeter, Thorax and Abdomen Respiratory Inductance, Plethysmography (RIP), Effort, Flow, Snore Microphone	III	The study was conducted on eighty adults with suspected OSA. The NOX T3 has a sensitivity of 85% and a specificity of 89% [43].	A study aimed to evaluate the accuracy of the NOX T3 method in diagnosing OSA through random tests on patients [44].

**Table 2:** Comparison of hardware-based methods for OSA detection found in scientific research.

Article	Number of Sensors	Type of Sensors	Performance
[58]	2	Photoplethysmography and accelerometer	Sensitivity and precision of 90%
[59]	1	MFOS	Accuracy of 49.96%, sensitivity of 57.07%, specificity of 45.26%
[60]	1	Force-sensitive resistor	Accuracy rate of 91%
[61]	1	SPO2 sensor	Accuracy of 88%, sensitivity of 80%, specificity of 91%
[62]	1	Tracheal sound sensor	Sensitivity of 96.06% and specificity of 76.07%
[63]	1	ECG sensor	Accuracy of 88.2%

**Table 3:** Summary of software-based methods for OSA detection found in scientific research.

Article	Input	Method	Accuracy
[64]	Infrared video	Deep learning	83%
[66]	Snoring signals	Deep neural network	74.27%
[68]	ECG signal	Multiscaled neural network	90.64%
[69]	ECG signal	Deep neural network	97.21%
[70]	ECG signal	Deep learning	99%
[71]	Pulse transition time	Deep learning	92.64%
[72]	SPO2 signal	Random forest classifier	82.8%
[74]	ECG signal	Artificial neural network	87.3%
[76]	Nasal pressure signal	Convolutional neural network	96.6%
[77]	Pulse oximetry	Regression modeling	96.7%
[78]	Mandibular jaw movements	Artificial intelligence	86.6%
[79]	ECG signal	Deep learning	92.15%
[80]	EEG signal	EEG-MIL	69.3–73.4%
[81]	EEG signal	EEG-CLNet	1–5% better than baseline

## Data Availability

No data were collected in this research.
